# Designing one-step reverse transcriptase loop-mediated isothermal amplification for serotype O foot-and-mouth disease virus detection during the 2022 outbreak in East Java, Indonesia

**DOI:** 10.14202/vetworld.2023.1889-1896

**Published:** 2023-09-17

**Authors:** Eduardus Bimo Aksono, Mirni Lamid, Rimayanti Rimayanti, Iwan Sahrial Hamid, Mustofa Helmi Effendi, Fedik Abdul Rantam, Widjiati Widjiati, Mufasirin Mufasirin, Heni Puspitasari, Munawaroh Fitria, Nur Syamsiatul Fajar, Lucia Tri Suwanti, Nusdianto Nusdianto, Andi Hamim Zaidan, Yuta Kanai, Teguh Hari Sucipto

**Affiliations:** 1 Department of Veterinary Medicine, Faculty of Veterinary Medicine, Universitas Airlangga, Surabaya, 60115, Indonesia; 2 Institute of Life Science, Technology and Engineering, Universitas Airlangga, Surabaya, 60115, Indonesia; 3 Institute of Tropical Disease, Universitas Airlangga, Surabaya, 60115, Indonesia; 4 Department of Clinical Pathology, Faculty of Medicine, Universitas Airlangga, Surabaya, 60132, Indonesia; 5 Research Institute for Microbial Diseases, Osaka University, Suita, Osaka, 565-0871, Japan

**Keywords:** cow, East Java, foot-and-mouth disease virus, reverse transcriptase loop-mediated isothermal amplification, reverse transcriptase polymerase chain reaction, serotype O

## Abstract

**Background and Aim:**

Various methods can detect foot-and-mouth disease (FMD) in cows, but they necessitate resources, time, costs, laboratory facilities, and specific clinical specimen submission, often leading to FMD virus (FMDV) diagnosis delays. The 2022 FMD outbreak in East Java, Indonesia, highlighted the need for an easy, inexpensive, rapid, and accurate detection approach. This study aims to devise a one-step reverse transcriptase loop-mediated isothermal amplification (RT-LAMP) technique and phylogenetic analysis to detect the serotype O FMDV outbreak in East Java.

**Materials and Methods:**

Swab samples were collected from the foot vesicles, nasal secretions, and saliva of five suspected FMDV-infected cows in East Java between June and July 2022. The RT-LAMP design used hydroxy naphthol blue dye or SYBR Green I dye, with confirmatory analysis through reverse transcriptase polymerase chain reaction (RT-PCR) targeting 249 base pairs. PCR products underwent purification, sequencing, and nucleotide alignment, followed by phylogenetic analysis.

**Results:**

The RT-LAMP method using hydroxy naphthol blue dye displayed a positive reaction through a color shift from purple to blue in the tube. Naked-eye observation in standard light or ultraviolet (UV) light at 365 nm, with SYBR Green I stain, also revealed color change. Specifically, using SYBR Green I dye, UV light at 365 nm revealed a color shift from yellow to green, signifying a positive reaction. Nucleotide alignment revealed mutations and deletion at the 15^th^ sequence in the JT-INDO-K3 isolate from the East Java FMDV outbreak. Despite differing branches, the phylogenetic tree placed it in the same cluster as serotype O FMDV from Malaysia and Mongolia.

**Conclusion:**

JT-INDO-K3 exhibited distinctions from Indonesian serotype O FMDV isolates and those documented in GenBank. Then, the RT-LAMP method used in this study has a detection limit 10 times higher latter than the conventional RT-PCR limit, without any cross-reactivity among strains.

## Introduction

Indonesia has been declared by the World Organization for Animal Health as foot-and-mouth disease (FMD)-free since 1990. However, another outbreak of FMD in Indonesia emerged in May 2022 from newly purchased cows in Balungpanggang subdistrict, Gresik Regency. Symptoms included hypersalivation, appetite loss, rapid breathing, and elevated temperature [1]. Foot-and-mouth disease is highly contagious with substantial economic impact. Knight-Jones *et al*. [2] approximated FMD’s annual impact, including production losses and vaccination demand in endemic areas, at US $6.5–$21.0 billion. In addition, outbreaks can result in annual losses exceeding US $1.5 billion in FMD-free nations. Hence, prompt identification of FMD viruses (FMDVs) is crucial for disease control and minimizing cattle losses, and rapid and accurate FMDV diagnosis is vital for effective disease management [3]. The disease stems from infection by FMDV, an *Aphthovirus* prototype within the *Picornaviridae* family. Foot-and-mouth disease virus particles contain a single positive-sense RNA genome copy (around 8300 nucleotides in length) [4]. The virus induces FMD, causing vesicles on the feet, mouth, tongue, and teats of cloven-hoofed animals, such as cows, sheep, goats, and pigs [3]. Foot-and-mouth disease virus is highly contagious, warranting reporting to the Office International des Epizooties [5].

Type A, O, and C FMDV phylogenetic trees primarily rely on the *VP1* gene’s nucleotide sequence, with *VP1*-based phylogenetic grouping correlating with classical serological classification [6–8]. Furthermore, the *VP1*-encoded nucleotide sequence is pivotal for FMDV characterization and phylogenetic analysis of capsid constituents [9–11]. According to Jamal *et al*. [4], FMDV comprises seven serotypes (O, A, C, Asia-1, SAT-1, SAT-2, and SAT-3). SAT serotypes are prevalent in sub-Saharan Africa, Type C in the Indian subcontinent, and Type Asia-1 in South Asia. Type A and Type O are widely distributed in most of Africa, South Asia, the Far East (excluding Type A), and South America. At present, Europe, North and Middle America, Greenland, Australasia, and Oceania are FMD-free [9]. Nonetheless, serotype O is frequently isolated from specimens exhibiting positive clinical symptoms and is suspected to have caused the Indonesian FMD outbreak in 2022.

Foot-and-mouth disease virus can be controlled through vaccination and early detection despite its infectious nature. Vaccination for one serotype offers limited or no protection against others, emphasizing the need to identify the correct vaccine for epidemic control [4, 12, 13]. Yoon *et al*. [14] outlined conventional FMDV detection methods, including antigen-specific enzyme-linked immunosorbent assay, cytopathic effect observation in cultures [3], reverse transcriptase polymerase chain reaction (RT-PCR) [15], and real-time quantitative polymerase chain reaction (qPCR) [16, 17]. These tests demand resources, time, costs, laboratory facilities, and special clinical specimen delivery, leading to FMDV diagnosis delays [18]. Given Indonesia’s challenging geography, swift and accurate diagnosis of suspected FMDV cases is imperative during outbreaks, particularly as the virus has a high contagion potential. One promising method under development is nucleic acid amplification through reverse transcriptase loop-mediated isothermal amplification (RT-LAMP) [3, 19]. This approach is used for detecting influenza A virus, Newcastle disease virus, and respiratory and reproductive disease syndrome viruses in pigs [20–22] and has been used in India to detect FMDV infection [14].

Rapid detection, vaccination programs, and observation of genomic and geographic shifts in serotype O FMDV are crucial preventive endeavors considering its widespread and swift dissemination. The present study aimed to develop the RT-LAMP method and genomic sequences, contributing to efforts to prevent and comprehend the diversity and evolution of serotype O FMDV. The method’s exceptional sensitivity and specificity could contribute to gaining vital insights essential for domestic and global FMDV control, potentially paving the way for commercial applications.

## Materials and Methods

### Ethical approval

This study was approved by the Animal Care and Use Committee (ACUC) Faculty of Veterinary Medicine, Universitas Airlangga, with approval number 1.KEH.1 August 06, 2022.

### Study period and location

The study was conducted during June and July 2022. The samples were collected from East Java, Indonesia, and processed at Institute of Tropical Disease, Universitas Airlangga.

### Samples

Fourteen swab samples were obtained from foot vesicles, nasal secretions, and saliva of five suspected FMDV-infected cows. Collected in separate vials with transport medium (Biobase Diagnostic Technology, Co., Ltd., Shandong), the samples were dispatched to the Institute of Life Science, Technology, and Engineering, Universitas Airlangga, and subsequently stored at −20°C. Reverse transcriptase loop-mediated isothermal amplification design using primers, following Farooq *et al*. [23], polymerase chain reaction (PCR) confirmation and phylogenetic analysis were performed at the Institute of Tropical Disease, Universitas Airlangga.

### One-step RNA extraction

To an aliquot of XpressAmp™ Lysis Buffer (Promega, Wisconsin, USA), 1-thioglycerol was added to reach a 1% (v/v) concentration. A sample lysate was formed by combining a VTM/UTM sample in a 1:1 ratio with freshly prepared XpressAmp™ Lysis Buffer containing 1% 1-thioglycerol (XpressAmp™ Direct Amplification Reagents, Cat#A8880, Promega). After pipetting and 10 min of incubation at 25 °C, the prepared sample lysates were used for subsequent PCR amplification.

### Reverse transcriptase loop-mediated isothermal amplification

The final 25 μL of the RT-LAMP mix comprised the following: 2.5 μL of ThermoPol reactive buffer (New England Bio Labs Inc., Beverly, MA, USA); 1 μL of MgSO_4_ (100 mM); 2 μL of dNTP set (10 mM; Fermentas); 5 μL of betaine (5 M; Sigma-Aldrich, St. Louis, MO, USA); 2 μL of nuclease-free water; 1 μL of hydroxy naphthol blue (3 mM; Sigma-Aldrich); 1 μL of Bst DNA polymerase Lg fragment (New England Bio Labs Inc.); 0.2 μL of AMV reverse transcriptase (New England Bio Labs Inc.,); 1 μL each of forward outer primers (CATCCTCACCACCCGTAAC) and backward outer primers (GACACCTTTGTGGTCGGTC), each at a 5 pmol concentration; 1 μL each of forward inner primers (GGAAGTGTTCGGTCCGCT CACTTTTCCCAGTCAAG CGTTGGAG) and backward inner primers (CAGAGTTGTGCAGGC AGAACGGTTTTA ACGTCCGA ATGAGTCACTG), each at a 50 pmol concentration; 1 μL each of forward loop primers (GGAGTCACATACGGGTACG) and backward loop primers (CACCTCTTCGACTGGGTC), each at a 25 pmol concentration; and 5 μL of RNA obtained from sample extraction. This mixture was incubated at 60°C for 15–60 min in a water bath. Detection of RT-LAMP results using hydroxy naphthol blue dye (Sigma-Aldrich; CAS: 63451-35-4) involved naked-eye observation for color changes in natural light or with assistance from ultraviolet (UV) light at 365 nm. When using SYBR Green I (Life Technologies, USA), the dye was applied after the RT-LAMP process in a 1:1 ratio between RT-LAMP product and SYBR Green I. Color changes were only detectable under UV light at 365 nm [24].

### Reverse transcriptase polymerase chain reaction

The samples subjected to RT-LAMP analysis were also assessed using RT-PCR for comparative purposes. Reverse transcriptase polymerase chain reaction followed the everTra Ace qPCR RT Master Kit protocol with gDNA Remover from Toyobo. For the final 10 μL mix, 2 μL of DN Master with gDNA Remover, 1 μL of Random Primer, 2 μL of nuclease-free water, 2 μL of 5× reverse transcriptase Master Mix II, and 3 μL of RNA sample were combined. Incubation was performed at 65°C for 1 min, 37°C for 15 min, 42°C for 30 min, and 98°C for 5 min. Following the reverse transcriptase procedure, amplification was conducted with a 12.5 μL mix consisting of 2× PCR Master mix Solution (*i*-Taq) *(*iNtRon, Gyeonggi-do, Republic of Korea), 1 μL of primer forward DHP13 (10 nmol; GTGACTGAACTGCTTTACCGCAT), 1 μL of primer reverse NK61 (10 nmol; GACATGTCCTCCTGCATCTG), 2.5 μL of nuclease-free water, and 3 μL of cDNA sample. The PCR reaction was performed using a Bioer Thermal cycler (Bioer Technology, Hangzhou, Japan) with the following temperature cycle: initial denaturation at 96°C for 30 s; 40 cycles of denaturation at 94°C for 15 s, annealing at 60°C for 30 s, and extension at 68°C for 30 s; and a final extension cycle at 68°C for 3 min [25, 26]. Electrophoresis of PCR results was performed on 2% agarose gel with TBE1×(Promega) and RedSafe (*i*NtRon, Gyeonggi-do, Republic of Korea) agarose gel dye. The electrophoresis was performed with Mupid-eXu (Mupid CO., LTD, Tokyo, Japan) at 100 V for 35 min, using a Nexmark™ 100 bp DNA marker (Nex™ Diagnostics, Gyeonggi-do, Korea) as the weight marker for the 249 bp PCR amplification target.

### Sequencing and phylogenetic analysis

Following RT-LAMP and RT-PCR analysis, the samples were subjected to phylogenetic analysis to confirm their serotype O FMDV isolate status. The PCR product from one sample (JT-INDO-K3) was forwarded to the Apical Scientific Laboratory (PT. Genetics Science Indonesia) with DHP13 primers [25, 26]. Analysis of the constructed phylogenetic tree was achieved using detected FMDV data through BLAST (https://blast.ncbi.nlm.nih.gov/Blast.cgi), along with references to whole genomes of FMDV serotype O isolates from GenBank.

### Measurement of RT-LAMP sensitivity and specificity

To assess RT-LAMP and RT-PCR detection limits, 10-fold serial dilutions of all samples (ranging from 3.25 × 10^5^ to 3.25 × 10^−1^ ng^/^mL) were prepared for sensitivity analysis. For RT-LAMP specificity evaluation, 14 swab samples from foot vesicles, nasal secretions, and saliva of 5 cows suspected of FMDV infection in East Java, including one FMDV Asia-1, one FMDV A, and one negative control (Table-1), were analyzed.

**Table 1:** Result of serotype O FMDV detection in swab samples confirmed with RT-LAMP and RT-PCR.

Pathogen	Strain	Sample type	RT-LAMP	RT-PCR
FMDV	O	Foot vesicles	5/5	5/5
		Nasal secretion	5/5	5/5
		Saliva	4/4	4/4
	Asia-1	Foot vesicles	0/0	0/0
		Nasal secretion	0/0	0/0
		Saliva	0/0	0/0
	A	Foot vesicles	0/0	0/0
		Nasal secretion	0/0	0/0
		Saliva	0/0	0/0

RT-LAMP=Reverse transcriptase loop-mediated isothermal amplification, RT-PCR=Reverse transcriptase polymerase chain reaction, FMDV=Foot-and-mouth disease virus

## Results and Discussion

### One-step RT-LAMP

**Figure-1:**
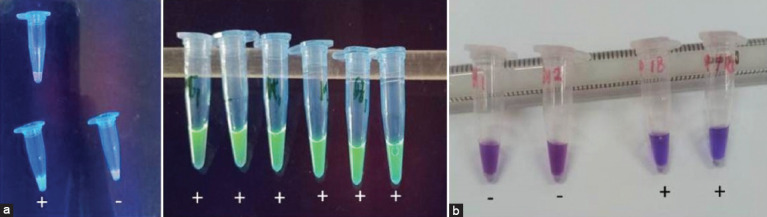
One-step reverse transcriptase loop-mediated isothermal amplification products from serotype O foot-and-mouth disease virus. (a) Using SYBR Green I dye (green color showing positive reaction); (b) Using hydroxy naphthol blue dye (blue color showing positive reaction).

In the one-step RT-LAMP approach, suspected FMDV presence in samples from foot vesicles, nasal secretions, and saliva was indicated by color changes. Using SYBR Green I dye with UV lighting at 365 nm, a yellow tube turning green denoted a positive FMDV result, whereas turning orange indicated a negative outcome (Figure-1a). With hydroxy naphthol blue dye, the one-step RT-LAMP method showed results through naked-eye observation in natural light. Positive FMDV reactions caused the purple tube to turn blue, whereas a lack of color change signaled a negative outcome (Figure-1b).

### Reverse transcriptase polymerase chain reaction

**Figure-2:**
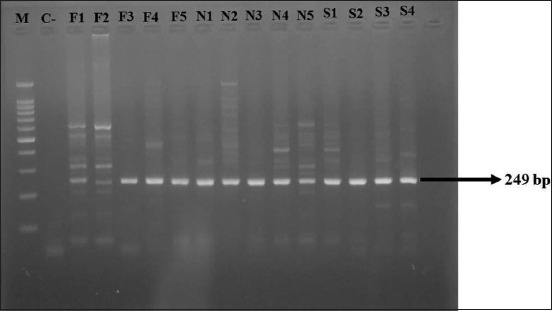
Reverse transcriptase polymerase chain reaction products from serotype O foot-and-mouth disease virus to electrophoresis gel 2% (249 bp). M=Marker 100 bp, C=Negative control, Samples F (foot visceral)=F1, F2, F2, F3, F4, F5 (positive), Samples N (secret nasal)=N1, N2, N3, N4, N5 (positive); S (Saliva)=S1, S2, S3, S4 (positive).

Swab samples from the foot vesicles, nasal secretions, and saliva of five cows suspected of FMDV infection in East Java were subjected to RT-LAMP using serotype O FMDV primers (F3, B3, FIP, BIP, FLP, and BLP), followed by confirmation through RT-PCR with *VP1* primers (DHP13-F and NK61-R). Both methods indicated positive FMDV identification in all swab samples, including foot vesicles (5/5), nasal secretions (5/5), and saliva (4/4) (Table-1). Reverse transcriptase polymerase chain reaction products exhibited a 249 bp target band with a 100 bp marker (Figure-2).

**Figure-3:**
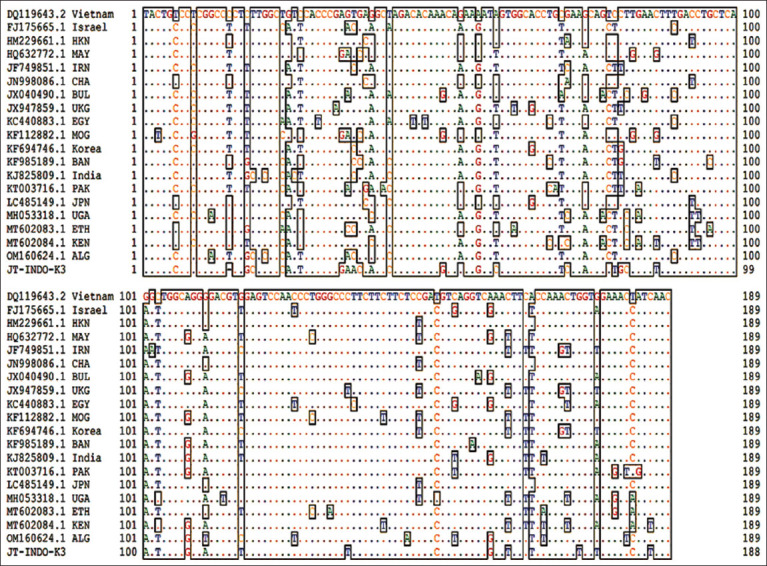
Nucleotide alignment of VP1 for serotype O foot-and-mouth disease virus.

**Figure-4:**
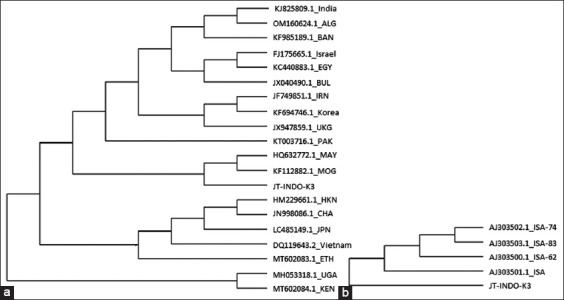
(a) Phylogenetic tree of VP1 for foot-and-mouth disease virus (FMDV) serotype O from some countries; (b) serotype O FMDV from Indonesia.

Nucleotide alignment results revealed that isolate JT-INDO-K3, which was associated with the East Java outbreak, displayed mutations and a 15^th^ nucleotide sequence deletion (Figure-3). Phylogenetic tree analysis (Figure-4a) comparing positive samples from East Java (JT-INDO-K3) with serotype O FMDV references from GenBank, such as those from Vietnam (Dq119643.2), Israel (FJ175665.1), Hong Kong (HM229661.1), Malaysia (HQ632772.1), Iran (JF749851.1), China (JN998086.1), Bulgaria (JX040490.1), United Kingdom (JX947859.1), Egypt (KC440883.1), Mongolia (KF112882.1), Korea (KF694746.1), Bangladesh (KF985189.1), India (KJ825809.1), Pakistan (KT003716.1), Japan (LC485149.1), Uganda (MH053318.1), Ethiopia (MT602083.1), Kenya (MT602084), and Algeria (OM160624.1), showed that serotype O FMDV isolates found during the June–July 2022 East Java outbreak clustered with those from Malaysia and Mongolia, despite differing branches. In contrast, comparison with serotype O FMDV isolates from Indonesia archived in GenBank, such as ISA-62 (AJ303500), ISA-74 (AJ303501), ISA-83 (AJ303502), and ISA (AJ503503), showed distinction from JT-INDO-K3 (Figure-4b).

The virus discussed here originated from a FMD outbreak in East Java during June and July 2022. Collection of virus samples was based on veterinary diagnoses of cows exhibiting FMD symptoms, including hypersalivation, excessive nasal secretion, appetite loss, mild temperature elevation, and vesicle formation on the feet and mouth. These manifestations align with the clinical symptoms reported by Sobhy *et al*. [27] that include hypersalivation and excessive nasal secretion progressing from mucoid to mucopurulent discharge covering the muzzle [27]. In addition, pyrexia reaching 40°C for 1–2 days and the occurrence of vesicles on the tongue, hard palate, dental pad, lips, gum, muzzle, coronary band, and interdigital space are observed. Vesicles are particularly likely on teats, especially in lactating cows, whereas calves usually die before displaying vesicles.

Examinations were conducted on five cows suspected of FMD in East Java using the RT-LAMP method, designed for cost-effective, rapid, and precise field detection, particularly for serotype O FMDV using a previously published primer set [28]. In the RT-LAMP process using hydroxy naphthol blue dye, a color shift from purple to blue indicates a positive reaction, whereas no change signifies a negative reaction. This color change can be perceived with the naked-eye under natural light or with the assistance of UV light at 365 nm [28]. Alternatively, with SYBR Green I dye, RT-LAMP analysis only manifests color alteration under UV light at 365 nm. A green hue in the yellow tube indicates a positive reaction, whereas an orange tint indicates negativity [24].

Reverse transcriptase loop-mediated isothermal amplification products from samples of the 2022 East Java FMD outbreak, gathered from foot vesicles (5/5), nasal secretion (5/5), and saliva (4/4), led to successful identification of serotype O FMDV in all instances. The outcomes were subsequently verified using the established gold standard FMDV detection method, RT-PCR, with recognized primers for serotype O FMDV [25]. Reverse transcriptase polymerase chain reaction also identified serotype O FMDV in every sample. This study provides robust evidence that RT-LAMP is a highly promising on-site detection technique that is straightforward, swift, and precise. This aligns with findings from Zhang *et al*. [19] and Lim *et al*. [29], highlighting the potential of RT-LAMP for FMDV detection, necessitating only a basic incubator, such as a heating block or water bath, and a process completion time of 15–60 min at a constant 60°C temperature. Fundamentally, the RT-LAMP method involves DNA synthesis through autocycling strand displacement facilitated by the Bst DNA polymerase large fragment. The combination of AMV reverse transcriptase and Bst DNA polymerase enables simultaneous reverse transcriptase reactions and DNA amplification within the same tube [19, 28]. The FMDV RT-LAMP test serves as a diagnostic tool with diagnostic specificity exceeding 99% and sensitivity of 79% [30].

On confirming that the June–July 2022 FMD outbreak in East Java was attributable to serotype O FMDV, PCR products with 249 bp underwent purification and sequencing to assess the potential presence of mutations. The *VP1* gene region of serotype O FMDV detected in East Java was contrasted with sequences of serotype O previously documented in GenBank from various countries, including Indonesia. Nucleotide alignment results indicated that JT-INDO-K3 isolates exhibited not only mutations but also a deletion at the 15^th^ nucleotide position. Furthermore, phylogenetic tree analysis of JT-INDO-K3 isolates revealed that despite divergent branches, these isolates clustered alongside Malaysian and Mongolian counterparts. In addition, JT-INDO-K3 isolates differed from previously reported Indonesian serotype O FMDV strains in GenBank.

The findings from this investigation suggest that despite having been recognized as FMD-free and acknowledged by the World Animal Health Organization since 1990 [1], Indonesia is no longer free from FMD. Analogous to the Malaysian scenario, occurrences of FMD cases in Peninsular Malaysia were linked to the FMD outbreak in Thailand due to their border proximity [31–33]. Hence, it is plausible that the East Java outbreak may have arisen from illicit animal distribution and direct contact with infected cattle in communal grazing areas situated near the Malaysian border. Furthermore, unlawful animal trade or the import of cattle into Indonesia, particularly East Java, could potentially be the primary source or a contributing factor to the outbreak. Thus, a comprehensive disease control initiative should encompass all border regions, not just in Malaysia but also other neighboring countries, and necessitate the enhancement of animal quarantine measures.

### Comparison of RT-LAMP and RT-PCR sensitivity

Positive RT-LAMP results were characterized by the emergence of diverse bands of varying sizes, as observed by agarose electrophoresis. Amplification through RT-LAMP exhibited a distinct ladder-like banding pattern (Figure-2). To assess the sensitivity and detection limits of RT-LAMP and RT-PCR, both techniques were applied to evaluate identical FMDV O samples. Illustrated in Figure-2, the detection limit of RT-PCR was 3.25 ng/mL. Conversely, the detection limit of RT-LAMP was approximately 3.25 × 10^−1^ ng^/^mL, representing a 10-fold increase compared with the conventional RT-PCR limit.

### Evaluation of FMDV RT-LAMP specificity

Analysis through agarose gel electrophoresis affirmed that only FMDV O RNA yielded a distinct positive reaction with RT-LAMP. Notably, no instances of cross-reactivity were observed with the FMDV A or FMDV Asia-1 samples. Conversely, healthy tissues produced negative reactions (Figures-1 and 2).

## Conclusion

The one-step RT-LAMP method, based on the *VP1* gene region and subsequently validated through RT-PCR, effectively detected a serotype O FMDV outbreak in East Java. This study revealed that the detection limits of RT-PCR and RT-LAMP were 3.25 ng/mL and approximately 3.25 × 10^−1^ ng^/^mL, respectively, with the latter being 10-fold higher than the conventional RT-PCR limit, without any cross-reactivity among strains. Nucleotide alignment revealed mutations and deletion at the 15^th^ nucleotide sequence. In contrast, phylogenetic tree analysis demonstrated that isolates from JT-INDO-K3, identified during the 2022 East Java outbreak, clustered alongside Malaysian and Mongolian serotype O FMDV isolates, despite differing branches. Notably, JT-INDO-K3 isolates differed from previously reported Indonesian serotype O FMDV strains archived in GenBank.

## Authors’ Contributions

EBA, ML, RR, ISH, MHE, and FAR: Concept and study design, data acquisition, and writing and critically reviewing the manuscript. WW, MM, MF, LTS, NN, AHZ, and YK: Concept and study and critical review of the manuscript. HP, NSF, and THS: Data acquisition and critical review of the manuscript. All authors have read, reviewed, and approved the final manuscript.

## References

[1] Sudarsono R.P (2022). Epidemiological study of suspected occurrence of foot and mouth disease in Lamongan Regency. J. Basic. Med. Vet.

[2] Knight-Jones T.J, McLaws M.M, Rushton J (2017). Foot-and-mouth disease impact on smallholders - what do we know, what don't we know and how can we find out more?. Transbound. Emerg. Dis.

[3] Wong C.L, Yong C.Y, Ong H.K, Ho K.L, Tan W.S (2020). Advances in the diagnosis of foot-and-mouth disease. Front. Vet. Sci.

[4] Jamal S.M, Belsham G.J (2018). Molecular epidemiology, evolution and phylogeny of foot-and-mouth disease virus. Infect. Genet. Evol.

[5] Kök S.A, Üstün S, Sezgin H.T (2023). Diagnosis of ruminant viral diseases with loop-mediated isothermal amplification. Mol. Biotechnol.

[6] El Nahas A.F, Salem S.A (2020). Meta-analysis of genetic diversity of the VP1 gene among the circulating O, A, and SAT2 serotypes and vaccine strains of FMD virus in Egypt. J. Vet. Res.

[7] Diab E, Bazid A.I, Fawzy M, El-Ashmawy W.R, Fayed A.A, El-Sayed M.M (2019). Foot-and-mouth disease outbreaks in Egypt during 2013–2014:Molecular characterization of serotypes A, O, and SAT2. Vet. World.

[8] Abu-Elnaga H.I, Rizk S.A, Daoud H.M, Mohamed A.A, Mossad W, Gamil M.A, Soudy A.F, El-Shehawy L.I (2020). Comparative nucleotide sequencing of the VP1 capsid gene of recent isolates of foot-and-mouth disease virus serotype O from Egypt. Arch. Virol.

[9] Belsham G.J, Kristensen T, Jackson T (2020). Foot-and-mouth disease virus:Prospects for using knowledge of virus biology to improve control of this continuing global threat. Virus Res.

[10] Najafi H, FallahMehrabadi M.H, Hosseini H, Ziafati Kafi Z, Modiri Hamdan A, Ghalyanchilangeroudi A (2020). The first full genome characterization of an Iranian foot and mouth disease virus. Virus Res.

[11] Bae S, Li V, Hong J, Kim J.N, Kim H (2021). Phylogenetic and evolutionary analysis of foot-and-mouth disease virus A/ASIA/Sea-97 lineage. Virus Genes.

[12] Sanson R.L, Rawdon T.G, van Andel M, Yu Z (2022). Modelling the field personnel resources to control foot-and-mouth disease outbreaks in New Zealand. Transbound. Emerg. Dis.

[13] Van Andel M, Tildesley M.J, Gates M.C (2021). Challenges and opportunities for using national animal datasets to support foot-and-mouth disease control. Transbound. Emerg. Dis.

[14] Yoon S.Y, Kang S.K, Lee H.B, Oh S.H, Kim W.S, Li H.S, Bok J.D, Cho C.S, Choi Y.J (2020). Enhanced efficacy of immunization with a foot-and-mouth disease multi-epitope subunit vaccine using mannan-decorated inulin microparticles. Tissue Eng. Regen. Med.

[15] Khan S, Ali Shah S.A, Jamal S.M (2021). Evaluation of sandwich enzyme-linked immunosorbent assay and reverse transcription polymerase chain reaction for the diagnosis of foot-and-mouth disease. Intervirology.

[16] Hole K, Nfon C (2019). Foot-and-mouth disease virus detection on a handheld real-time polymerase chain reaction platform. Transbound. Emerg. Dis.

[17] El Bagoury G.F, Elhabashy R, Mahmoud A.H, Hagag N.M, El Zowalaty M.E (2022). Development and evaluation of one-step real-time RT-PCR assay for improved detection of foot-and-mouth disease virus serotypes circulating in Egypt. J. Virol. Methods.

[18] Lippi G, Betsou F, Cadamuro J, Cornes M, Fleischhacker M, Fruekilde P, Neumaier M, Nybo M, Padoan A, Plebani M, Sciacovelli L, Vermeersch P, von Meyer A, Simundic A.M, Working Group for Preanalytical Phase (WG-PRE) European Federation of Clinical Chemistry and Laboratory Medicine (EFLM) (2019). Preanalytical challenges-time for solutions. Clin. Chem. Lab. Med.

[19] Zhang J, Hou Q, Ma W, Chen D, Zhang W, Wubshet A.K, Ding Y, Li M, Li Q, Chen J, Dai J, Wu G, Zhang Z, Zaberezhny A.D, Pejsak Z, Tarasiuk K, Khan M.U, Wang Y, He J, Liu Y (2022). A naked-eye visual reverse transcription loop-mediated isothermal amplification with sharp color changes for potential pen-side test of foot-and-mouth disease virus. Viruses.

[20] Storms S.M, Shisler J, Nguyen T.H, Zuckermann F.A, Lowe J.F (2023). RT-LAMP as diagnostic tool for influenza-a virus detection in swine. Vet. Sci.

[21] Selim K, Adel A, Eid S, Shahein M (2022). Development of real time reverse transcription loop-mediated isothermal amplification assay for rapid detection of genotype VII of Newcastle disease viruses. Br. Poult. Sci.

[22] Gao X, Liu X, Zhang Y, Wei Y, Wang Y (2020). Rapid and visual detection of porcine deltacoronavirus by recombinase polymerase amplification combined with a lateral flow dipstick. BMC Vet. Res.

[23] Farooq U, Latif A, Irshad H, Ullah A, Zahur A.B, Naeem K, Khan S.U, Ahmed Z, Rodriguez L.L, Smoliga G (2015). Loop-mediated isothermal amplification (RT-LAMP):A new approach for the detection of foot-and-mouth disease virus and its sero-types in Pakistan. Iran. J. Vet. Res.

[24] Ge A, Liu F, Teng X, Cui C, Wu F, Liu W, Liu Y, Chen X, Xu J, Ma B (2022). A Palm Germ-Radar (PaGeR) for rapid and simple COVID-19 detection by reverse transcription loop-mediated isothermal amplification (RT-LAMP). Biosens. Bioelectron.

[25] Ranjitha H.B, Dhanesh V.V, Hosamani M, Sreenivasa B.P, Jabeen U, Biswal J. K, Saravanan P, Sanyal A, Bhanuprakash V, Basagoudanavar S.H (2023). Thermostable negative-marker foot-and-mouth disease virus serotype o induces protective immunity in guinea pigs. Appl. Microbiol. Biotechnol.

[26] Deepak P.R, Saravanan P, Biswal J.K, Basagoudanavar S.H, Dechamma H.J, Umapathi V, Sreenivasa B.P, Tamilselvan R.P, Krishnaswamy N, Zaffer I, Sanyal A (2019). Generation of acid resistant virus like particles of vaccine strains of Foot-and-Mouth Disease Virus (FMDV). Biologicals.

[27] Sobhy N.M, Bayoumi Y.H, Mor S.K, El-Zahar H.I, Goyal S.M (2018). Outbreaks of foot and mouth disease in Egypt:Molecular epidemiology, evolution and cardiac biomarkers prognostic significance. Int. J. Vet. Sci. Med.

[28] Ghaith D.M, Abu Ghazaleh R.A (2021). Carboxamide and N-alkylcarboxamide additives can greatly reduce non specific amplification in loop-mediated isothermal amplification for Foot-and-Mouth Disease Virus (FMDV) using Bst 3.0 polymerase. J. Virol. Methods.

[29] Lim D.R, Kim H.R, Chae H.G, Ku B.K, Nah J.J, Ryoo S, Wee S.H, Lee C, Lyoo Y.S, Park C.K (2020). Probe-based real-time reverse transcription loop-mediated isothermal amplification (RRT-LAMP) assay for rapid and specific detection of foot-and-mouth disease virus. Transbound. Emerg. Dis.

[30] Bath C, Scott M, Sharma P.M, Gurung R.B, Phuentshok Y, Pefanis S, Colling A, Balasubramanian N.S, Firestone S.M, Ungvanijban S, Ratthanophart J, Allen J, Rawlin G, Fegan M, Rodoni B (2020). Further development of a reverse-transcription loop-mediated isothermal amplification (RT-LAMP) assay for the detection of foot-and-mouth disease virus and validation in the field with use of an internal positive control. Transbound. Emerg. Dis.

[31] Chanchaidechachai T, Saatkamp H, de Jong M, Inchaisri C, Hogeveen H, Premashthira S, Buamitoup N, Prakotcheo R, van den Borne B. H (2022). Epidemiology of foot-and-mouth disease outbreaks in Thailand from 2011 to 2018. Transbound. Emerg. Dis.

[32] Blacksell S.D, Siengsanan-Lamont J, Kamolsiripichaiporn S, Gleeson L.J, Windsor P.A (2019). A history of FMD research and control programmes in Southeast Asia:Lessons from the past informing the future. Epidemiol. Infect.

[33] Dian N.D, Rahim M.A, Chan S, Idris Z.M (2022). Non-human primate malaria infections:A review on the epidemiology in Malaysia. Int. J. Environ. Res. Public Health.

